# 2-(4-Amino­phen­yl)-1,3-benzoxazole

**DOI:** 10.1107/S160053680801653X

**Published:** 2008-06-07

**Authors:** Yuan Qu, Shi-lei Zhang, Lei Teng, Xian-you Xia, Yong Zhang

**Affiliations:** a School of Chemical and Materials Engineering, Huangshi Institute of Technology, Huangshi 435003, People’s Republic of China

## Abstract

In the title mol­ecule, C_13_H_10_N_2_O, the dihedral angle between the benzoxazole ring system and the benzene ring is 11.8 (1)°. In the crystal structure, mol­ecules are linked by inter­molecular N—H⋯N hydrogen bonds and π⋯π inter­actions [centroid–centroid distance = 3.6560 (15) Å] to form a two-dimensional network.

## Related literature

For related literature, see: Prudhomme *et al.* (1986 ▶); Vinsova *et al.* (2005 ▶).
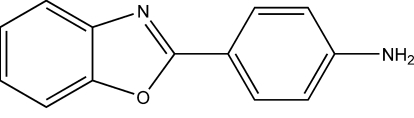


## Experimental

### 

#### Crystal data

C_13_H_10_N_2_O
*M* *_r_* = 210.23
Monoclinic, 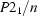

*a* = 4.1461 (3) Å
*b* = 19.5420 (12) Å
*c* = 12.7705 (8) Å
β = 95.243 (1)°
*V* = 1030.38 (12) Å^3^
*Z* = 4
Mo *K*α radiation
μ = 0.09 mm^−1^
*T* = 298 (2) K
0.30 × 0.20 × 0.15 mm

#### Data collection

Bruker SMART CCD diffractometer
Absorption correction: multi-scan (*SADABS*; Sheldrick, 2003 ▶) *T*_min_ = 0.974, *T*_max_ = 0.987
4628 measured reflections
1902 independent reflections
1315 reflections with *I* > 2σ(*I*)
*R*_int_ = 0.086

#### Refinement

*R*[*F* ^2^ > 2σ(*F* ^2^)] = 0.065
*wR*(*F* ^2^) = 0.151
*S* = 1.08
1902 reflections
151 parameters
2 restraints
H atoms treated by a mixture of independent and constrained refinement
Δρ_max_ = 0.17 e Å^−3^
Δρ_min_ = −0.22 e Å^−3^

### 

Data collection: *SMART* (Bruker, 2001 ▶); cell refinement: *SAINT-Plus* (Bruker, 2001 ▶); data reduction: *SAINT-Plus*; program(s) used to solve structure: *SHELXS97* (Sheldrick, 2008 ▶); program(s) used to refine structure: *SHELXL97* (Sheldrick, 2008 ▶); molecular graphics: *SHELXTL* (Sheldrick, 2008 ▶); software used to prepare material for publication: *SHELXTL*.

## Supplementary Material

Crystal structure: contains datablocks global, I. DOI: 10.1107/S160053680801653X/lh2630sup1.cif

Structure factors: contains datablocks I. DOI: 10.1107/S160053680801653X/lh2630Isup2.hkl

Additional supplementary materials:  crystallographic information; 3D view; checkCIF report

## Figures and Tables

**Table 1:** Hydrogen-bond geometry (Å, °)

*D*—H⋯*A*	*D*—H	H⋯*A*	*D*⋯*A*	*D*—H⋯*A*
N2—H2*B*⋯N1^i^	0.868 (10)	2.174 (12)	3.028 (3)	168 (3)

Symmetry code: (i) 
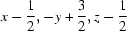
.

**Fig. 1.**
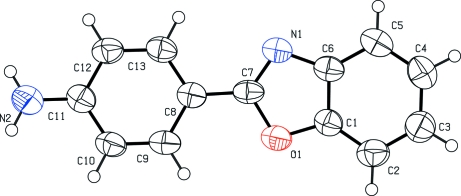
Molecular structure of (I), showing the atom-numbering scheme. Displacement ellipsoids for non-H atoms are drawn at the 50% probability level.

**Fig. 2.**
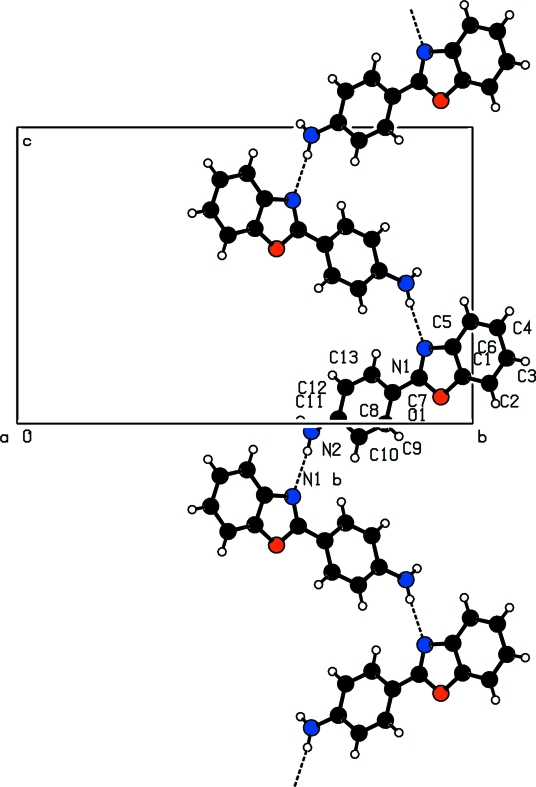
Part of the crystal packing showing the formation of the [101] chains linked by by N—H···N hydrogen bonds shown as dashed lines.

**Fig. 3.**
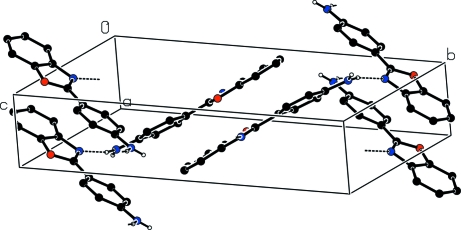
Part of the crystal packing showing the formation of the two-dimensional layers formed by by N—H···N hydrogen bonds shown as dashed lines and π···π interactions. For clarity, H atoms not involved in the motif have been omitted.

## supplementary crystallographic information

### Comment

The benzoxazole rings sytem is one of the most common heterocycles in medicinal chemistry. Previous reports revealed that substituted benzoxazoles possess diverse chemotherapeutic activities including antibiotic, antimicrobial, antiviral and antitumor activities (Prudhomme *et al.*, 1986; Vinsova *et al.*, 2005). With this mind, the title compound, (I), was prepared in a series of syntheses to produce new benzoxazole derivatives, and we report the crystal stucture herein.

The molecular structure of (I) is illustrated in Fig. 1. In (I), the benzixazole rings system is not co-planar with the benzene ring, the dihedral angle being 11.8 (1)°. In the crystal structure, molecules are linked by N2—H2B···N1^i^ hydrogen bonds (symmetry code: (i) *x* -1/2, 3/2 - *y*, *z* - 1/2) into a one-dimensional chains along [101] (Fig. 2). Neighbouring chains are further linked into a two-dimensional network by π..π interactions with Cg1···Cg2(-1+x, y, z) = 3.6560 (15) Å where Cg1 and Cg2 are the centroids defined by the ring atoms O1/C1/C6/N1/C7  and C1-C6 respectivley. There are no significant interactions between the adjacent layers.

### Experimental

All reagents and solvents were used as obtained without further purification. 4-aminobenzoic acid (13.7 g, 0.1 mol) and 2-aminophenol (10.9 g, 0.1 mmol) were mixed together with polyphosphoric acid (50 g) and heated to 493 K under N_2_ atmosphere for 4 h. The reaction mixture was cooled to room temperature and poured into 10% K_2_CO_3_ solution. The precipitate was filtered under reduced pressure. Brown crystals were obtained by recrystallization from acetone-water.Yield: 88%; Analysis calculated for C_13_H_10_N_2_O: C 74.29, H 4.76, N 13.33%; found: C 74.26, H 4.78, N 13.35%.

### Refinement

All the aromatic H atoms were located at the geometrical positions with C—H=0.93 Å(aromatic), and the *U*_iso_ values were set 1.2 times of their carrier atoms. H2A and H2B were found in difference Fourier maps and refined with the constraint of *N*—*H*=0.86 Å (amine) and *U*_iso_(H)=1.2*U*_eq_(N).

### Crystal data

**Table:**

C_13_H_10_N_2_O	*F*_000_ = 440
*M**_r_* = 210.23	*D*_x_ = 1.355 Mg m^−^^3^
Monoclinic, *P*2_1_/*n*	Mo *K*α radiation λ = 0.71073 Å
Hall symbol: -P 2yn	Cell parameters from 1089 reflections
*a* = 4.1461 (3) Å	θ = 2.6–22.6º
*b* = 19.5420 (12) Å	µ = 0.09 mm^−^^1^
*c* = 12.7705 (8) Å	*T* = 298 (2) K
β = 95.243 (1)º	Block, brown
*V* = 1030.38 (12) Å^3^	0.30 × 0.20 × 0.15 mm
*Z* = 4	

### Data collection

**Table:**

Bruker SMART CCD diffractometer	1902 independent reflections
Radiation source: fine-focus sealed tube	1315 reflections with *I* > 2σ(*I*)
Monochromator: graphite	*R*_int_ = 0.086
*T* = 298(2) K	θ_max_ = 25.5º
φ and ω scans	θ_min_ = 1.9º
Absorption correction: multi-scan(SADABS; Sheldrick, 2003)	*h* = −4→5
*T*_min_ = 0.974, *T*_max_ = 0.987	*k* = −23→17
4628 measured reflections	*l* = −14→15

### Refinement

**Table:**

Refinement on *F*^2^	Secondary atom site location: difference Fourier map
Least-squares matrix: full	Hydrogen site location: inferred from neighbouring sites
*R*[*F*^2^ > 2σ(*F*^2^)] = 0.065	H atoms treated by a mixture of independent and constrained refinement
*wR*(*F*^2^) = 0.151	*w* = 1/[σ^2^(*F*_o_^2^) + (0.0636*P*)^2^ + 0.0374*P*] where *P* = (*F*_o_^2^ + 2*F*_c_^2^)/3
*S* = 1.09	(Δ/σ)_max_ < 0.001
1902 reflections	Δρ_max_ = 0.17 e Å^−^^3^
151 parameters	Δρ_min_ = −0.22 e Å^−^^3^
2 restraints	Extinction correction: none
Primary atom site location: structure-invariant direct methods	

### Special details

**Table:**

Geometry. All e.s.d.'s (except the e.s.d. in the dihedral angle between two l.s. planes) are estimated using the full covariance matrix. The cell e.s.d.'s are taken into account individually in the estimation of e.s.d.'s in distances, angles and torsion angles; correlations between e.s.d.'s in cell parameters are only used when they are defined by crystal symmetry. An approximate (isotropic) treatment of cell e.s.d.'s is used for estimating e.s.d.'s involving l.s. planes.
Refinement. Refinement of *F*^2^ against ALL reflections. The weighted *R*-factor *wR* and goodness of fit *S* are based on *F*^2^, conventional *R*-factors *R* are based on *F*, with *F* set to zero for negative *F*^2^. The threshold expression of *F*^2^ > σ(*F*^2^) is used only for calculating *R*-factors(gt) *etc*. and is not relevant to the choice of reflections for refinement. *R*-factors based on *F*^2^ are statistically about twice as large as those based on *F*, and *R*- factors based on ALL data will be even larger.

### Fractional atomic coordinates and isotropic or equivalent isotropic displacement parameters (Å^2^)

**Table:**

	*x*	*y*	*z*	*U*_iso_*/*U*_eq_	
O1	1.1291 (4)	0.93054 (9)	0.09030 (12)	0.0548 (5)	
N1	1.0571 (5)	0.89549 (10)	0.25399 (15)	0.0508 (6)	
C8	0.8394 (6)	0.82440 (13)	0.10509 (18)	0.0471 (6)	
C7	1.0044 (6)	0.88277 (13)	0.15444 (18)	0.0472 (6)	
C13	0.6743 (6)	0.77900 (13)	0.16499 (19)	0.0522 (7)	
H13	0.6617	0.7882	0.2360	0.063*	
C11	0.5492 (6)	0.70536 (14)	0.01598 (19)	0.0511 (7)	
C1	1.2748 (6)	0.97814 (13)	0.15893 (18)	0.0488 (7)	
N2	0.4179 (7)	0.64627 (14)	−0.02632 (19)	0.0746 (8)	
H2B	0.430 (7)	0.6376 (13)	−0.0924 (9)	0.090 (1)*	
H2A	0.300 (6)	0.6220 (12)	0.0115 (19)	0.090 (1)*	
C6	1.2309 (6)	0.95673 (13)	0.25916 (18)	0.0487 (7)	
C12	0.5301 (6)	0.72124 (13)	0.1221 (2)	0.0561 (7)	
H12	0.4183	0.6923	0.1638	0.067*	
C9	0.8540 (6)	0.80871 (14)	−0.00072 (18)	0.0529 (7)	
H9	0.9622	0.8382	−0.0428	0.064*	
C2	1.4362 (7)	1.03717 (14)	0.1363 (2)	0.0630 (8)	
H2	1.4620	1.0504	0.0676	0.076*	
C5	1.3567 (7)	0.99539 (15)	0.3444 (2)	0.0615 (8)	
H5	1.3315	0.9819	0.4130	0.074*	
C10	0.7122 (6)	0.75067 (14)	−0.04423 (19)	0.0556 (7)	
H10	0.7254	0.7415	−0.1152	0.067*	
C3	1.5567 (7)	1.07529 (15)	0.2214 (2)	0.0664 (8)	
H3	1.6650	1.1159	0.2103	0.080*	
C4	1.5205 (7)	1.05458 (16)	0.3237 (2)	0.0640 (8)	
H4	1.6088	1.0812	0.3795	0.077*	

### Atomic displacement parameters (Å^2^)

**Table:**

	*U*^11^	*U*^22^	*U*^33^	*U*^12^	*U*^13^	*U*^23^
O1	0.0637 (12)	0.0592 (12)	0.0423 (10)	0.0044 (9)	0.0097 (8)	0.0024 (9)
N1	0.0542 (13)	0.0567 (15)	0.0422 (12)	0.0052 (11)	0.0075 (9)	0.0029 (10)
C8	0.0499 (15)	0.0475 (16)	0.0443 (14)	0.0116 (12)	0.0062 (11)	0.0034 (12)
C7	0.0491 (15)	0.0520 (17)	0.0419 (14)	0.0135 (13)	0.0115 (11)	0.0073 (12)
C13	0.0545 (16)	0.0579 (19)	0.0453 (15)	0.0110 (14)	0.0119 (12)	−0.0013 (13)
C11	0.0529 (16)	0.0502 (17)	0.0502 (15)	0.0121 (13)	0.0053 (12)	−0.0028 (13)
C1	0.0528 (16)	0.0470 (16)	0.0469 (15)	0.0099 (13)	0.0059 (11)	−0.0013 (12)
N2	0.097 (2)	0.0717 (19)	0.0584 (15)	−0.0086 (15)	0.0221 (14)	−0.0112 (14)
C6	0.0493 (15)	0.0537 (17)	0.0439 (14)	0.0127 (13)	0.0082 (11)	0.0023 (12)
C12	0.0601 (17)	0.0587 (19)	0.0518 (16)	0.0057 (14)	0.0173 (13)	0.0044 (13)
C9	0.0594 (17)	0.0594 (18)	0.0410 (14)	0.0089 (14)	0.0103 (11)	0.0072 (12)
C2	0.071 (2)	0.063 (2)	0.0568 (17)	0.0044 (16)	0.0167 (14)	0.0039 (15)
C5	0.0651 (18)	0.072 (2)	0.0468 (16)	0.0091 (16)	0.0051 (13)	−0.0044 (14)
C10	0.0680 (18)	0.0591 (19)	0.0408 (14)	0.0067 (15)	0.0111 (13)	−0.0006 (13)
C3	0.067 (2)	0.063 (2)	0.070 (2)	−0.0023 (15)	0.0143 (15)	−0.0052 (16)
C4	0.0577 (18)	0.069 (2)	0.0643 (19)	0.0032 (16)	0.0024 (14)	−0.0143 (15)

### Geometric parameters (Å, °)

**Table:**

O1—C7	1.374 (3)	N2—H2B	0.868 (10)
O1—C1	1.379 (3)	N2—H2A	0.859 (10)
N1—C7	1.294 (3)	C6—C5	1.387 (3)
N1—C6	1.395 (3)	C12—H12	0.9300
C8—C13	1.392 (3)	C9—C10	1.372 (3)
C8—C9	1.392 (3)	C9—H9	0.9300
C8—C7	1.445 (4)	C2—C3	1.373 (4)
C13—C12	1.368 (3)	C2—H2	0.9300
C13—H13	0.9300	C5—C4	1.379 (4)
C11—N2	1.367 (4)	C5—H5	0.9300
C11—C10	1.388 (3)	C10—H10	0.9300
C11—C12	1.399 (3)	C3—C4	1.389 (4)
C1—C6	1.374 (3)	C3—H3	0.9300
C1—C2	1.378 (4)	C4—H4	0.9300
			
C7—O1—C1	104.26 (18)	C5—C6—N1	131.3 (2)
C7—N1—C6	104.6 (2)	C13—C12—C11	120.6 (2)
C13—C8—C9	117.4 (2)	C13—C12—H12	119.7
C13—C8—C7	120.0 (2)	C11—C12—H12	119.7
C9—C8—C7	122.4 (2)	C10—C9—C8	121.4 (2)
N1—C7—O1	114.6 (2)	C10—C9—H9	119.3
N1—C7—C8	127.7 (2)	C8—C9—H9	119.3
O1—C7—C8	117.7 (2)	C3—C2—C1	115.9 (3)
C12—C13—C8	121.7 (2)	C3—C2—H2	122.0
C12—C13—H13	119.2	C1—C2—H2	122.0
C8—C13—H13	119.2	C4—C5—C6	117.5 (2)
N2—C11—C10	121.1 (2)	C4—C5—H5	121.2
N2—C11—C12	121.0 (2)	C6—C5—H5	121.2
C10—C11—C12	117.9 (2)	C9—C10—C11	121.0 (2)
C6—C1—C2	123.9 (2)	C9—C10—H10	119.5
C6—C1—O1	107.4 (2)	C11—C10—H10	119.5
C2—C1—O1	128.7 (2)	C2—C3—C4	121.6 (3)
C11—N2—H2B	119.6 (19)	C2—C3—H3	119.2
C11—N2—H2A	118 (2)	C4—C3—H3	119.2
H2B—N2—H2A	122 (3)	C5—C4—C3	121.5 (3)
C1—C6—C5	119.5 (3)	C5—C4—H4	119.3
C1—C6—N1	109.2 (2)	C3—C4—H4	119.3
			
C6—N1—C7—O1	0.1 (3)	C7—N1—C6—C5	−179.6 (3)
C6—N1—C7—C8	178.5 (2)	C8—C13—C12—C11	1.1 (4)
C1—O1—C7—N1	−0.1 (3)	N2—C11—C12—C13	177.1 (3)
C1—O1—C7—C8	−178.7 (2)	C10—C11—C12—C13	−1.4 (4)
C13—C8—C7—N1	9.5 (4)	C13—C8—C9—C10	−0.2 (4)
C9—C8—C7—N1	−166.4 (2)	C7—C8—C9—C10	175.9 (2)
C13—C8—C7—O1	−172.1 (2)	C6—C1—C2—C3	0.1 (4)
C9—C8—C7—O1	12.0 (3)	O1—C1—C2—C3	179.7 (2)
C9—C8—C13—C12	−0.3 (4)	C1—C6—C5—C4	0.4 (4)
C7—C8—C13—C12	−176.4 (2)	N1—C6—C5—C4	179.9 (2)
C7—O1—C1—C6	0.1 (2)	C8—C9—C10—C11	−0.1 (4)
C7—O1—C1—C2	−179.6 (2)	N2—C11—C10—C9	−177.6 (3)
C2—C1—C6—C5	−0.7 (4)	C12—C11—C10—C9	0.9 (4)
O1—C1—C6—C5	179.6 (2)	C1—C2—C3—C4	0.9 (4)
C2—C1—C6—N1	179.7 (2)	C6—C5—C4—C3	0.5 (4)
O1—C1—C6—N1	0.0 (3)	C2—C3—C4—C5	−1.2 (4)
C7—N1—C6—C1	−0.1 (3)		

### Hydrogen-bond geometry (Å, °)

**Table:**

*D*—H···*A*	*D*—H	H···*A*	*D*···*A*	*D*—H···*A*
N2—H2B···N1^i^	0.868 (10)	2.174 (12)	3.028 (3)	168 (3)

Symmetry codes: (i) *x*−1/2, −*y*+3/2, *z*−1/2.
